# Effect of Different Plasticizers on Thermal, Crystalline, and Permeability Properties of Poly(3–hydroxybutyrate–co−3–hydroxyhexanoate) Films

**DOI:** 10.3390/polym14173503

**Published:** 2022-08-26

**Authors:** Yousof Farrag, Luis Barral, Oreste Gualillo, Danny Moncada, Belén Montero, Maite Rico, Rebeca Bouza

**Affiliations:** 1 NEIRID Group (Neuroendocrine Interactions in Rheumatology and Inflammatory Diseases), IDIS (Instituto de Investigación Sanitaria de Santiago de Compostela), Santiago University Clinical Hospital, Building C, Travesía da Choupana s/n, 15706 Santiago de Compostela, Spain; 2 Grupo de Polímeros, Departamento de Física y Ciencias de la Tierra, Escuela Universitaria Politécnica, Serantes, Universidade da Coruña, Avda. 19 de Febrero s/n, 15471 Ferrol, Spain

**Keywords:** PHBH, cast films, biopolymers, plasticizers, thermal properties, permeability properties

## Abstract

Poly(3−hydroxybutyrate−co−3−hydroxyhexanoate) (PHBH) films were prepared using a cast film technique. Dioxane was chosen over other polymer solvents as it resulted in homogenous films with better morphology. Several plasticizers with different molecular weights and concentrations were added to the biopolymer solution prior to casting. Thermal, crystalline, and permeability properties were analyzed by thermogravimetric analysis (TGA), differential scanning calorimetry (DSC), X−ray diffraction (XRD), and both water vapor and oxygen transmission rate analysis. In general, the addition of plasticizers decreased the glass transition temperature (T_g_), cold crystallization temperatures (T_cc_), melting temperatures, as well as crystallinity degrees and increased the crystallite sizes and water vapor and oxygen transmission rates. The use of isosorbide and low-molecular-weight poly(ethylene glycol) (PEG) lowered the Tg around 30 °C at the highest used concentration, also being the most effective in increasing the crystallite size. When considering isosorbide and low-molecular-weight poly(ethylene glycol) (PEG) as very good plasticizers for PHBH, the question of which plasticizer to use strongly relies on the desired PHBH application.

## 1. Introduction

Biopolymers derived from sustainable sources such as plants, bacteria, and animal sources are in the center of attention of scientific research during the last years for environmental, sustainable, and biocompatibility reasons. Looking at the actual situation of plastic waste management, the biodegradability of biopolymers at the end of their life cycle presents great importance that situates biopolymers as a potential alternative to conventional plastics in many industries, especially plastics for short-term use [1]. The biocompatibility of many biopolymers also makes them very promising candidates for many biomedical applications, including wound healing, tissue engineering, drug delivery, medical devices, and scaffold fabrication [2,3]. 

Polyhydroxyalkanoates (PHAs) are linear polyester biopolymers produced by a wide variety of bacteria to store carbon and energy [4]. They are easy to extract using some organic solvents followed by precipitation in water. They are thermoplastics, i.e., they can be repeatedly softened by heating and hardened again on cooling and, in general, can be processed using the conventional processing equipment of plastics [5]. They are biocompatible and biodegradable biopolymers with many applications in the biomedical field as well as in the packaging industry [6,7,8].

PHAs can be produced as homopolymers or copolymers with different lengths of the side chain depending on the microorganism and the cultivation conditions. Poly(3−hydroxybutyrate) (PHB) is the most common PHA homopolymer that presents good mechanical and thermal properties as well as good processability. Despite that, PHB is known to be very brittle, which limits its application [7]. Copolymers of PHB with other hydroxyalkanoic acids with longer side chains show significant improvements in mechanical and thermal properties [9]. Poly(3-hydroxybutyrate-co−3-hydroxyhexanoate) (PHBH) is a medium-chain-length PHA copolymer that has been studied in the last years for different applications [10]. The hydroxyhexanoate fraction of the copolymer is known to decrease the polymer’s crystallinity and the glass transition temperature, resulting in a more flexible polymer with a larger window of processing [11]. PHBH also showed better mechanical and processability properties than the widely studied small-chain PHA, poly(3−hydroxybutyrate−co−3−hydroxyvalerate) (PHBV). It also showed better biocompatibility with a wide range of cell cultures than both PHB and PHBV [12,13,14,15]. 

Despite these promising data, the way of many biopolymers, including PHAs, into industry faces some difficulties such as high pricing compared to that of conventional plastics and metals; less availability of information about the structure–properties relationship; and the processing and mechanical limitations of some of them. Several ways are being followed to improve the mechanical and processability limitations of biopolymers in specific and polymers in general. Examples of these improvement approaches are chemical modifications, physical blending with other polymers, addition of fillers, and the use of plasticizers [5,16,17,18]. Plasticizers are materials with small molecular weight for promoting plasticity, flexibility, processability, and reducing brittleness [19]. Plasticizers enhance the processability by lowering the glass transition temperature (Tg), resulting in the reduction of the polymer’s stiffness and brittleness, allowing polymer mixing and extrusion at lower temperatures, less pressure, and shorter time. The use of plasticizers also has more possible impacts on related physical properties of the plasticized polymer such as melting and crystallization temperatures, crystallinity, and permeability to water vapor and other gases [20].

Compared with other PHAs like PHB and PHBV, there is much less published research around PHBH. This paper investigates the fundamental structure–properties relationship of plasticized PHBH and aims to explore the effect of the usage of several molecules as plasticizers on the thermal, crystallinity, and gas permeability properties of PHBH films. These properties were studied analyzing the data obtained from TGA, DSC, XRD, and water vapor and oxygen transmission rates analysis. The effect of changing the plasticizer concentration on these properties was analyzed too. The PHBH films were prepared by film casting techniques. The parameters of the film casting, including the polymer solvent, the temperature of solvent evaporation, and the concentration of the polymer solution, were optimized to get smooth, homogeneous, and translucent films. 

Two novel plasticizers were selected in this study: isosorbide and glycerol diacetomonolaurate (GDAL). Isosorbide is an innovative green biodegradable nontoxic plasticizer used recently for other biopolymers [21,22,23,24]. GDAL is also a nontoxic food-grade acetylated monoglyceride plasticizer that is reported as an effective plasticizer for PLA [20,25]. Poly(ethylene glycol) (PEG) with different molecular weights was also chosen as it is among the most investigated plasticizers, especially for biopolymers, besides being widely recognized as biocompatible materials [26].

## 2. Materials and Methods

### 2.1. Materials and Reagents

Poly(3-hydroxybutyrate−co−3−hydroxyhexanoate) with 11% of hydroxyhexanoate (HH) content (PHBH) and molecular weights of 500–600 kDa was kindly gifted by Kaneka Co, Brussels, Belgium. Dichloromethane, chloroform, dimethyl sulfoxide, 1,4 dioxane, poly(ethylene glycol) (PEG) with molecular weights of 200, 400, and 600 g/mol, were purchased from Scharlau, Barcelona, Spain. Poly(ethylene glycol) (PEG) with molecular weight of 100 kDa was purchased from Sigma Aldrich, Bavaria, Germany. Dimethyl carbonate was purchased from Alfa Aesar, Kandel, Germany. Isosorbide (C6H10O4) with a purity of 98% was purchased from Acros Organics, Geel, Belgium. Glycerol diacetomonolaurate (Biocizer ®) with purity ≥ 95% was kindly gifted by Riken Vitamin Co, Tokyo, Japan. All chemicals were used without further purification. 

### 2.2. Preparation of The Films of PHBH

The PHBH films were prepared using the solution casting method. In general, 0.8 g of PHBH granules were weighed and completely dissolved in 20 mL of polymer solvent using mechanical stirring in addition to heat when required. The plasticizers were added to the PHBH solution and mixed until complete miscibility or dissolution. The plasticizers were added in different weight percentages of the polymer content according to the concentrations listed in Table 1. A fixed volume of 20 mL of the polymer solution (with or without plasticizer) was poured in flat-bottom glass petri dish with a diameter of 90 mm and left to dry for several hours. The drying was performed at different temperatures, avoiding an air stream. 

### 2.3. Thermogravimetric Analysis (TGA) 

Thermogravimetric analysis was performed using a PerkinElmer TGA − 4000 microbalance with a ceramic sample pan (Massachusetts, USA). Approximately 10–15 mg of the PHBH films were heated from 50 °C to 500 °C at 10 °C min^−1^ with a 20 mL min^−1^ nitrogen flow. The thermogravimetric curves and their first derivatives, DTG curves, were recorded in each test. The onset temperature of degradation (T_o_), the maximum temperature of degradation (T_max_), and the end temperature of degradation (T_end_) were measured from DTG curves. T_max_ was measured at the peak maximum of the DTG curves while T_o_ and T_end_ were measured at degradation rates of 0.05 and 0.01, respectively.

### 2.4. Differential Scanning Calorimetry (DSC)

Differential scanning calorimetry was performed by a Pyris−1 DSC (Perkin Elmer, Norwalk, CT, USA) under a nitrogen atmosphere. The samples were heated from −30 °C to 200 °C at 10 °C min^−1^, then were cooled to −30 °C and subsequently heated again to 200 °C at the same rate. Three replicates were performed for each sample, and the data are presented as the average value.

### 2.5. X-ray Diffraction (XRD) 

X-ray diffraction patterns were recorded in a diffractometer D5000 Siemens (Karlsruhe, Germany). The equipment operated at a voltage of 45 kV and a current of 30 mA and used copper K−alpha (Cu Kα) radiation with an average wavelength of λ (Kα) = 0.15 nm (1.54 Å). The aperture used was 0.6 mm. Diffractograms were registered in the angular region of 2θ from 5° to 40°, at room temperature, with a step time of 4s and an angular increment of 0.005°.

### 2.6. Water Vapor Transmission Rate

The water vapor transmission rate (WVTR) was measured with a Permatran−W, 1/50 G, Mocon equipment, (Mocon Inc., Minnesota, USA). Smooth and uniform parts of the PHBH film samples with a thickness of 80 μm were selected. Masks with a 5 cm^2^ test area were used for putting samples in the cell of the equipment at 37.8 °C. Relative humidities of 5% and 40% were applied to the two sides of the films as the driving force of the test. WVTR was calculated by counting passed water molecules through the film every 30 min. Passed molecules were carried to the counting part of the equipment by nitrogen as a carrying gas which was purged continuously during the test. The tests were continued until a steady line for a transmission rate under a continuous mode (WVTR, in g m^−2^ day^−1^) was obtained.

### 2.7. Oxygen Transmission Rate

The oxygen transmission rate (OTR) was measured with an OX-TRAN, 1/50 G, Mocon equipment (Mocon Inc., Minnesota, USA). Smooth and uniform parts of the PHBH film samples with a thickness of 80 μm were selected. Masks with a 5 cm^2^ test area were used for putting samples in the cell of equipment. The temperature of the cell was 23 °C. One side of the film was exposed to a dry oxygen flow, relative humidity of 0.5%, with a pressure of 30 psi. Then, the diffusion process of oxygen molecules to the film started. OTR was calculated by counting passed molecules through the film every 30 min. Passed molecules were carried to the counting part of the equipment by dry nitrogen carrying gas which was purged continuously with the pressure of 30 psi during the test. The tests were continued a steady line for a transmission rate under a continuous mode (OTR, in cm^3^ m^−2^ day^−1^) was obtained.

## 3. Results

### 3.1. Preparation of the Films of PHBH

In order to obtain homogeneous films of PHBH, many formulations were tested by changing several parameters, including the organic solvent, the concentration of the polymer solution, the total volume of the polymer solution, and the temperature of evaporation.

Firstly, chloroform and dichloromethane were used as they are excellent solvents for PHBH besides being the most used solvents for dissolving other PHAs such as PHB and PHBV. The granules of PHBH were easily soluble in both solvents at room temperature, making a transparent solution with noticeable viscosity that increased with increasing polymer concentration. The obtained films after casting were nonhomogeneous with translucent and white zones, rough surfaces with bubble-like structure and large variations in thickness within the same film. These results were obtained repeatedly although the temperature of the solvent evaporation was changed from room temperature to 40, 60, and 90 °C. Changing the volume of the polymer solution from 10 to 20, 40, and 60 mL and the polymer concentration from 1 up to 8 *w*/*v*% did not show any improvement of this nonhomogeneous morphology.

Better and more homogeneous films were obtained using dioxane or dimethyl carbonate as solvents at different polymer concentrations and applying different temperatures for solvent evaporating. However, when using dimethyl carbonate, the films showed some wrinkles at the surface. Dimethyl sulfoxide (DMSO) is also a good solvent for PHBH, however, due to its high boiling point (189 °C), it requires relatively high temperatures and a long time to be evaporated. Furthermore, films obtained using DMSO were still nonhomogeneous. 

Films prepared using dioxane as the polymer solvent were smooth, homogenous, and translucent. Increasing the solvent evaporation temperatures from 40 up to 80 °C accelerated the evaporation of dioxane which has a boiling point of 101 °C. The prepared films were found to have the best visual morphology when the solvent was evaporated at 80 °C. Building on that, each of the films used for the rest of the experiments of this study was prepared by dissolving 0.8 g of PHBH in 20 mL of dioxane which was subsequently evaporated at 80 °C. The obtained films were around 80 µm in thickness, although this value can increase up to 100 µm when plasticizers are included to the formula. Table 1 resumes the plasticizers and their concentrations used for the preparation of the PHBH films. The comparisons between the different plasticizers were performed using PHBH films prepared with 10 and 20 % *w*/*w* calculated based on the polymer weight. Additional films with a plasticizer concentration of 50% were prepared in the case of using GDAL or isosorbide to evaluate whether the plasticizers maintained their functionality at such high concentration. Additional plasticizer concentrations were used for the TGA experiment for better clarification of the plasticizer concentration impact on the degradation behavior.

### 3.2. Thermogravimetric Analysis of The PHBH Films

**Figure 1:**
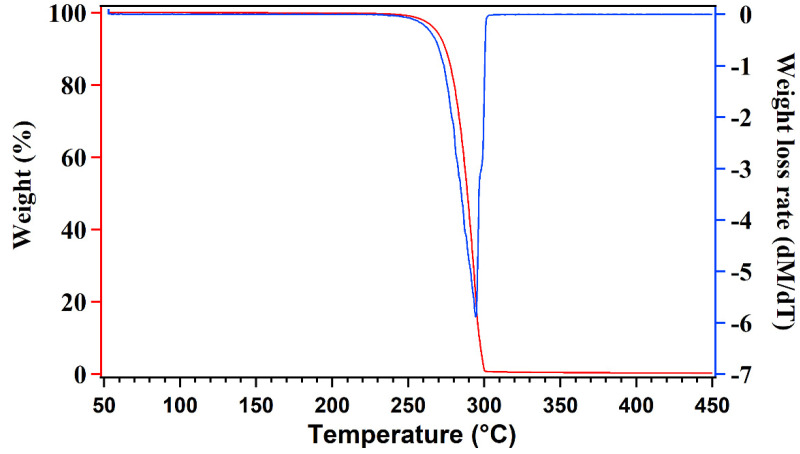
TGA (red) and DTG (blue) curves of P−0.

Thermogravimetric analysis (TGA) was realized to reveal the effect of the incorporation of plasticizers to the polymeric films on thermal degradation. The TGA and the DTG curves of the PHBH films are presented in Figures S1–S8. Table 2 resumes the TGA parameters of PHBH films. The degradation temperature of the PHBH films without plasticizer started at 262.7 °C (Figure 1). The rate of the degradation reached its maximum value at 291.6 °C. The polymer was completely degraded within only 38 °C at 301.1 °C. These values are in agreement with the values published by Hosoda et al. and Ivorra-Martinez et al. [27,28].

The T_o_ of the isosorbide plasticized films with concentrations higher than 5% was constant at 174.0 °C. This behavior of the isosorbide plasticized films is related to the onset of the degradation of the plasticizer itself at 170 °C. A small degradation step at around 250 °C can be seen in films with an isosorbide concentration higher than 20% which is attributed to the plasticizer-rich moiety of the films. Films plasticized with isosorbide also showed slightly higher T_max_ and lower degradation rates than the nonplasticized film. The interval degradation of the isosorbide plasticized films was 110 °C for the 5% plasticizer films and increased by increasing the isosorbide concentration to reach 171 °C for films with 50% plasticizer.

Although T_o_ of GDAL was 202.6 °C, PHBH films plasticized with GDAL did not start their degradation until 216.6 and 250.2 °C at 50% and 5% of plasticizer concentration, respectively. Small degradation shoulders started at around 305 °C and were noted for films plasticized with 20% GDAL or more, which is related to the degradation of the plasticizer-rich phase of the films.

Films plasticized with PEG with a molecular weight higher than 200 showed clear two steps of degradation behavior. The first step started at temperatures around 240 °C, T_max_ around 290 °C, and T_end_ at 310 °C. The second step of degradation showed T_o_ at around 355 °C, T_max_ between 400 and 410 °C, and T_end_ between 430 and 440 °C. First-step degradation happened at 70 °C, while the second one was around 80 °C. The first step of degradation was attributed to the degradation of the PHBH polymer which was shifted to lower temperature compared with that of the nonplasticized polymer. The second step of the film degradation represented the degradation of the plasticizer-rich moiety of the film as it was around the same temperatures of degradation of PEG. PEG 200 degradation happened at lower temperatures and started at 167 °C. This affected the onset temperatures of degradation of the films plasticized with it to be 231.7 °C at 10% and 217.0 °C at 20% of plasticizer concentration. These films also did not show the second degradation step that was noticed with the rest of the PEG plasticized films; however, they showed a little shoulder between 305 and 350 °C. Both the early degradation and the shoulder were related to the degradation behavior of the PEG 200 itself and were attributed to the plasticizer-rich moieties of the films.

In general, the use of a plasticizer altered the thermal degradation behavior mainly by slowing the degradation rate, lowering the onset of the degradation T_o_, and inducing two-step degradation in some cases. The increase in plasticizer concentration was associated with a further increase of the degradation intervals and lowering of the T_o_ too. Films plasticized with isosorbide showed the lowest T_o_ as the degradation of the isosorbide started earlier than with the other plasticizers used. 

### 3.3. DSC of The PHBH Films

**Figure 2:**
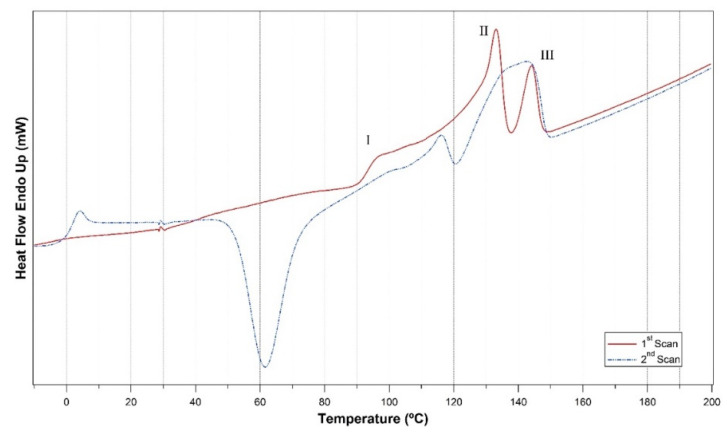
DSC curves of the first and second heating cycles of P−0.

The DSC technique was used to study the thermal properties of the PHBH films. Figure 2 shows the curves of the first and the second heating cycles of the PHBH film with no plasticizer, while those corresponding to the plasticized films are presented in Figures S9–S16. PHBH showed the typical multiple melting behavior as in other PHAs [12,29]. Three melting endotherms are clearly observed in the first heating cycle in Figure 2 and are named I, II, and III. The first endotherm I was attributed to the melting of the secondary lamellae, the second endotherm II to the melting of the primary lamellae, while the third endotherm III to the melting of the reorganized and/or thickened lamellae during the DSC heating process [12,30]. 

The shape and the position of the endothermic melting peaks depend on crystallization conditions such as temperature and the presence of additives that can alter the crystallization process. All films used for DSC analysis were cast under the same conditions, letting the dioxane to evaporate at 80 °C. For these conditions, the peaks of the endotherms I, II, and III during the first heating cycle were at temperatures T_I_, T_II_, and T_III_ of 96.5, 133.1, and 144.1 °C, respectively.

The second heating cycle revealed the T_g_ of the nonplasticized PHBH to be at 2.0 °C. An exothermic peak at around 62 °C could be observed due to cold crystallization. The melting behavior of the polymer showed considerable changes too with a smaller peak at the endotherm I; shifting of the endotherm II to a lower temperature accompanied by a decrease in the intensity; endotherm III became much broader and occupied the range of both endotherms II and III in the first heating cycle. The differences in the crystallization conditions outside (during the film casting) and inside the DSC invoked the differences in the shape and the position of the endothermic melting peaks between the first and the second heating cycles, as stated before. Table 3 details the positions of the melting peaks of the first heating cycle, shows the melting enthalpies of both heating cycles and T_g_ of each film.

The plasticized PHBH films showed significant changes in their melting and glass temperatures. The decrease of the melting temperatures T_II_ and T_III_ was proportional to the increase of the plasticizer concentration except for the PEG 100kDa plasticized films where T_II_ and T_III_ values did not show significant changes. T_I_ showed slight fluctuation with GDAL as the plasticizer and was stable at around 93 °C for the other plasticizers for most of the concentrations. The enthalpy of melting decreased as well by adding plasticizers and increasing their concentrations, indicating significant decreases of the crystallinity.

Increasing the plasticizer concentration caused a further decrease of T_g_ of the plasticized films. Using GDAL as the plasticizer caused a relatively slight decrease of the T_g_ to reach only −4.6 °C at the maximum concentration (50%). T_g_ reached less than −20 °C at plasticizer concentrations of 20% for isosorbide and PEG of low molecular weights. PEG 100kDa lowered the T_g_ to −9.6 °C at 10% and only to −0.7 °C at a 20% concentration. In case of isosorbide, the T_g_ value for the film with 50% was below −30 °C, which was beyond the cooling capabilities of the used DSC system and could not be registered. 

With the incorporation of the isosorbide and PEG of low molecular weights, there was a noticeable decrease in the cold crystallization temperatures (T_cc_). With isosorbide, T_cc_ decreased from 61.6 °C for neat P−0 film to 46.2, 41.4, and 22.8 °C for plasticizer concentrations of 10, 20, and 50%, while the decrease in T_cc_ was less pronounced with the PEG with low molecular weights. Increasing the molecular weight of the PEG from 200 to 400 resulted in lowering the T_cc_ at all used concentrations. T_cc_ of the films plasticized with PEG 600 were higher than those of the films plasticized with PEG 400 at 10% concentration, and even higher than those with PEG 200 at 20%. This might indicate less effectiveness of the PEG 600 as a plasticizer compared with that of the PEG with lower molecular weights. An initial decrease of the T_cc_ was noticed in films with 10% GDAL; however, there was a subsequent increase at 20% and 50% GDAL to 63.4 and 74.8 °C. The T_cc_ of films plasticized with PEG 100kDa could not be determined with precision due to the interference between the cold crystallization peaks with an endothermic peak that corresponded to the plasticizer melting. This melting peak indicated a possible phase separation and low compatibility with the polymer.

The integration of the plasticizer small molecules between the polymer chains increases its mobility, thus decreases the T_g_, T_cc_, and the melting temperatures. These in general are desirable effects of the plasticizers that result in decreasing the polymer brittleness and increasing their flexibility, processability, and distensibility. The exceptional behavior of the PEG 100kDa might be due to the high molecular weight of that plasticizer as it starts to separate the polymer chains at a low concentration, leading to lowering the T_g_; then, the large chains of such plasticizers start to interfere with the polymer chain mobility at higher concentrations, leading to increasing T_g_. The long PEG chains also did not allow a further decrease of the melting temperatures to happen when increasing its concentration.

### 3.4. X-ray Diffraction (XRD) of The PHBH Films

**Figure 3:**
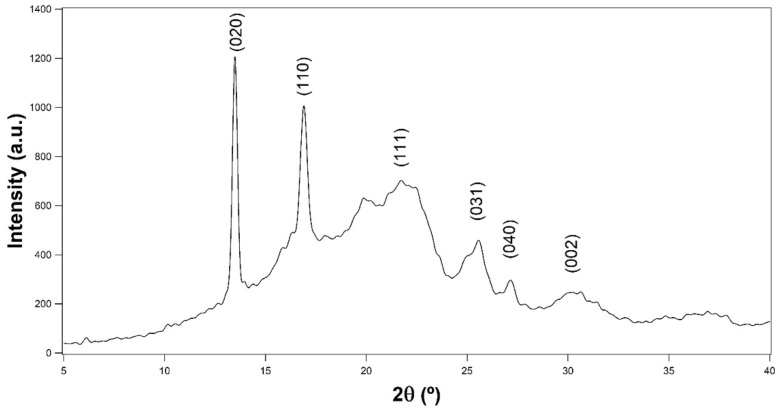
XRD pattern of P−0.

Medium-chain-length PHAs are known to have helix conformations forming an orthorhombic lattice with two molecules per unit cell [31]. The PHBH was reported with the same orthorhombic lattice system, P2_1_2_1_2_1_(D_2_^4^) (α = β = γ = 90°) with a = 5.76 Å, b = 13.20 Å and c = 5.96 Å [32]. Figure 3 shows the obtained diffraction pattern of the PHBH film with no plasticizer. The XRD diffractograms of the plasticized films are presented in Figures S17–20. The pattern shows the typical peaks at 2θ = 13.49°, 16.90°, 21.81°, 25.49°, 27.13°, and 30.31° that corresponds to the (020), (110), (111), (031), (040), and (002) reflections, respectively [32]. 

The addition of different plasticizers showed limited influence on the peak positions and the interplanar spacing (d spacing) of the PHBH films (Table 4). The d spacing and the lattice parameters a, b, and c showed very slight changes by the incorporation of the plasticizers. The noticeable change was found in the full width at half maximum of the peak (FWHM), where the incorporation of the plasticizers decreased their values. According to the Scherrer equation (Equation (1)), crystallite size is inversely proportional to the FWHM, as follows:

(1) $$D=k\lambda /\left(\beta cos\theta \right)$$

where D is the apparent crystallite size in nanometers, λ is the wavelength of the X-ray radiation, k is a shape factor constant with value of 0.9 in the calculations, β is FWHM in radians, and θ is the peak position in radians. 

The apparent crystallite sizes D_020_ of the PHBH films were calculated using the data of the strongest scattering intensity peak at 13.49° that corresponded to the (020) reflection (Table 4). The crystallite size of the pure PHBH film was 25.49 nm. This value increased always by increasing the plasticizer concentration. It is worth noting that isosorbide and PEG of low molecular weights, the plasticizers that shifted the T_g_ to the least value as tested by DSC, resulted also in the biggest crystallite size comparing with that of the other plasticizers at a given concentration. On the other hand, the use of PEG 100kDa resulted in a slight increase of the crystallite size, just 0.19 to 0.27 nm at 10 and 20% concentrations, respectively. The increase of the molecular weight of PEG from 200 to 600 also resulted in a slight increase of the crystal size (26.26, 26.64, and 26.58 nm at Mw 200, 400, and 600, respectively). The increase of the crystal size with increasing the molecular weight was higher at 20% PEG. The longer PEG chains might result in increasing the free volume, allowing for slightly increased crystal growth, with a more noticeable effect at higher PEG concentrations. There was no noticeable increase in crystallite size when using GDAL at 10%. Increasing the GDAL concentration increased the crystallite size by smaller values than with isosorbide and low molecular weight PEG. Such changes in the crystallite sizes seem also to be attributed to the changes in molecular mobility because of plasticizer incorporation. Isosorbide and PEG of low molecular weights seemed to offer higher molecular mobility than GDAL and PEG 100kDa, allowing for the growth of bigger crystallites. 

Diffraction intensity data, in the range of 2θ between 5° and 40°, were used for the calculations of the degree of crystallinity, dividing the area of the crystalline peaks by the area of both crystalline peaks and an amorphous halo. The obtained values of crystallinity presented in Table 4 show that the degree of crystallinity decreased as the plasticizer concentration increased. This is in good agreement with the tendency of the melting enthalpies obtained from the DSC analysis. It is worth noting that increasing PEG molecular weight did decrease the crystallinity at 10% PEG, however, the crystallinity increased at 20% PEG with the increment of the molecular weight. This might indicate that although the longer chain PEG might increase the free volume, it did not result better plasticization at already such high plasticizer concentration. 

### 3.5. Water Vapor and Oxygen Transmission Rates

The determination and the control of the transmission of water vapor and certain gases through polymeric films is of great importance especially for some applications such as the packaging industry. For example, controlling the passage of some gases through films used for food packaging is critical for keeping food fresh and healthy if possible. The effect of the incorporation of plasticizers and their concentrations on the WVTR and OTR was analyzed, and the results are presented in Table 5. The data show that the water vapor and the oxygen transmission rates of the nonplasticized PHBH film were 3.23 g m^−2^ day^−1^ and 14.75 cm^3^ m^−2^ day^−1^, respectively. These values increased significantly with increasing plasticizer content. As the plasticizer molecules penetrate in-between the polymer chains and increase the free volume of the polymer, this leads to increasing the diffusion and permeation rate of small molecules in general [33]. In addition to that, most plasticizers are hydrophilic and even hygroscopic in nature and tend to absorb water molecules, forming a hydrodynamic plasticizer–water complex, which favors water vapor passage furthermore [19].

Films prepared with GDAL as the plasticizer showed lower values of WVTR than PEG plasticized films did at most concentrations. This behavior might be attributed to the less hydrophilic nature of such acetylated glycerol fatty acid ester. WVTR of the films is dependent on matrix microstructures, crystallinity, and interaction between polymer components which subsequently affects the hydrophobicity of the film [34]. On the other side, the OTR values of these films were much higher than the values obtained with other plasticized films. This result, in addition to the low effectiveness of GDAL in both decreasing T_g_ (Table 3) and increasing the crystallite size (Table 4), may suggest reduced polymer-plasticizer compatibility.

The increase of the WVTR of the films plasticized with isosorbide was the lowest among most plasticized films at certain plasticizer concentrations despite its high hygroscopic nature. These films also showed a slight decrease in their OTR comparing to that of nonplasticized films. This behavior was reported before for maize starch films plasticized with isosorbide too. González et al. explained this behavior as a possible result of the high polarity and the steric hindrance produced by the plasticizer ring giving rise to a lower increase of the free volume and stiffer chains [35]. The interaction between the plasticizer and the polymer may also reduce the free hydroxyl groups, decrease hygroscopicity, and therefore decrease the interaction with water [36]. This seems to be a valid explanation considering that isosorbide was the least effective plasticizer in decreasing the degree of crystallinity of PHBH films (Table 4). Moreover, plasticization possibly induces a collapse of solid structures, giving denser matrices, which further inhibits diffusion of gas and decreases permeability of the films [37]. The gas transmission data as well the potential lowering of T_g_ and the increasing of the crystallite size suggest high PHBH compatibility with isosorbide and low-molecular-weight PEG plasticizers.

## 4. Conclusions

The effects of using different plasticizers in different concentrations on the thermal, structural, and permeability properties of PHBH films were analyzed. In general, the use of plasticizers increased the molecular mobility, which gave rise to considerable dropping of T_g,_ T_cc_, and melting points, lower degrees of crystallinity, bigger crystallite size, higher oxygen and water vapor transmission rates, and lower temperatures of thermal degradation. Isosorbide and low-molecular-weight PEG seemed to be more effective in lowering T_g_ and increasing the crystallite size. Despite that, isosorbide plasticized films did show the lowest increase of their WTVR, no increase of their OTR, and higher crystallinity. The use of GDAL significantly decreased the crystallinity; however, it was less effective in decreasing the T_g_ and increasing the crystallite size and produced films with excessively high OTR. These results may suggest poorer polymer–plasticizer compatibility than most of the tested plasticizers. Although PEG 100kDa still acted like a plasticizer, its high molecular weight and long chains hindered its functionality. The selection of the adequate plasticizer and its concentration are keys to controlling the properties of the desired final product. The produced films are of potential importance to a wide range of applications, especially the packaging industry and biomedicine.

## Figures and Tables

**Table 1:** PHBH films prepared with different plasticizers and different plasticizer concentrations.

Film ID	Plasticizer	Plasticizer w%
P−0	–	0
PG−05	GDAL	5
PG−10		10
PG−20		20
PG−30		30
PG−40		40
PG−50		50
PI−05	Isosorbide	5
PI−10		10
PI−20		20
PI−30		30
PI−40		40
PI−50		50
PP200−10	PEG 200	10
PP200−20		20
PP400−10	PEG 400	10
PP400−20		20
PP600−10	PEG 600	10
PP600−20		20
PP100k−10	PEG 100k	10
PP100k−20		20

**Table 2:** TGA parameters of PHBH films.

Film ID	T_o_ (°C)	T_max_ (°C)	T_end_ (°C)
P−0	262.7	291.6	301.1
PG−05	250.2	296.6	360.1
PG−10	243.4	296.0	361.3
PG−20	233.0	299.6	362.9
PG−30	236.1	300.1	388.1
PG−40	230.0	300.1	388.1
PG−50	216.6	289.0	388.1
PI−05	251.3	298.5	311.8
PI−10	174.0	295.6	316.8
PI−20	174.0	296.6	316.8
PI−30	174.0	297.3	320.1
PI−40	174.0	298.1	321.4
PI−50	174.0	301.7	330.2
PP200−10	231.7	289.9	398.6
PP200−20	217.0	290.2	414.2
PP400−10	^(1)^ 247.0/^(2)^ 325.6	^(1)^ 294.7/^(2)^ 389.6	^(1)^ 313.1/^(2)^ 434.1
PP400−20	^(1)^ 243.5/^(2)^ 325.4	^(1)^ 288.8/^(2)^ 397.7	^(1)^ 313.1/^(2)^ 438.7
PP600−10	^(1)^ 240.9/^(2)^ 328.8	^(1)^ 288.7/^(2)^ 402.7	^(1)^ 310.1/^(2)^ 437.6
PP600−20	^(1)^ 237.1/^(2)^ 328.8	^(1)^ 284.6/^(2)^ 407.8	^(1)^ 310.1/^(2)^ 440.8
PP100k−10	^(1)^ 248.4/^(2)^ 346.3	^(1)^ 287.5/^(2)^ 409.3	^(1)^ 301.9/^(2)^ 432.3
PP100k−20	^(1)^ 242.5/^(2)^ 364.4	^(1)^ 285.8/^(2)^ 404.6	^(1)^ 301.9/^(2)^ 432.3

^(1)^ Values for the first degradation step. ^(2)^ Values for the second degradation step.

**Table 3:** DSC parameters of PHBH films: first (Tm I), second (Tm II), and third (Tm III) melting temperature; melting enthalpies of the first (∆H_m1_) and second (∆H_m2_) heat cycles; cold crystallization temperature (T_cc_); glass transition temperature (T_g_).

Film ID	First Heat Cycle				∆H_m2_ (J/g)	T_cc_ (°C)	T_g_ (°C)
	T_m_ I (°C)	T_m_ II (°C)	T_m_ III (°C)	∆H_m1_ (J/g)			
P−0	96.5	133.1	144.1	43.4	36.4	61.6	2.0
PG−10	98.9	131.7	142.5	46.4	35.7	54.3	1.0
PG−20	92.9	125.1	135.7	32.4	28.2	63.4	−4.5
PG−50	95.0	121.9	131.2	32.0	11.8	74.8	−4.6
PI−10	95.9	130.4	140.6	39.3	31.2	46.2	−12.1
PI−20	93.4	127.5	138.1	34.6	24.1	41.4	−24.1
PI−50	92.5	123.9	134.8	29.0	20.6	22.8	–
PP200−10	96.4	130.7	140.9	37.1	38.7	59.9	−12.0
PP200−20	93.4	125.8	136.4	33.9	39.5	46.4	−26.2
PP400−10	94.4	130.4	142.1	32.5	29.8	50.6	−16.5
PP400−20	93.4	126.0	137.0	28.1	30.8	41.9	−24.6
PP600−10	93.5	129.7	140.6	30.5	36.8	58.6	−18.7
PP600−20	93.4	127.9	139.1	33.4	37.1	49.2	−28.2
PP100k−10	93.4	129.3	140.4	40.8	31.1	–	−9.6
PP100k−20	93.4	128.6	139.8	33.5	30.7	–	−0.7

**Table 4:** Crystallinity parameters of the PHBH films.

Film ID	Lattice Parameters (Å)			*d*_020_	Crystal Size Scherrer D_020_ (nm)	Crystallinity (%)
	a	b	c			
P−0	5.71	13.12	5.88	6.55	25.49	55.40
PG−10	5.70	13.11	5.87	6.55	25.46	46.25
PG−20	5.69	13.07	5.85	6.53	26.25	39.59
PG−50	5.69	13.07	5.84	6.53	27.31	31.75
PI−10	5.69	13.07	5.86	6.53	26.41	48.14
PI−20	5.68	13.07	5.86	6.53	27.33	45.09
PI−50	5.68	13.07	5.86	6.53	28.99	36.87
PP200−10	5.66	13.03	5.82	6.49	26.26	42.70
PP200−20	5.69	13.08	5.83	6.53	26.54	36.24
PP400−10	5.68	13.07	5.84	6.53	26.64	42.44
PP400−20	5.68	13.07	5.84	6.53	26.92	39.00
PP600−10	5.68	13.07	5.84	6.53	26.58	41.86
PP600−20	5.70	13.09	5.86	6.54	27.38	39.53
PP100k−10	5.69	13.08	5.84	6.53	25.68	44.56
PP100k−20	5.68	13.08	5.83	6.53	25.76	39.10

**Table 5:** Water vapor and oxygen transmission rates of PHBH films.

Film ID	Water Vapor Transmission Rate (g m^−2^ day^−1^)	Oxygen Transmission Rate (cm^3^ m^−2^ day^−1^)
P−0	3.23	14.75
PG−10	9.84	44.52
PG−20	13.73	67.94
PG−50	35.97	148.30
PI−10	6.69	12.41
PI−20	14.15	11.17
PI−50	30.05	11.27
PP200−10	14.97	17.17
PP200−20	27.82	27.36
PP400−10	8.90	27.67
PP400−20	38.71	32.01
PP600−10	12.71	27.66
PP600−20	54.67	31.375
PP100k−10	32.74	22.11
PP100k−20	36.83	25.98

## Data Availability

Not applicable.

## Supplementary Materials

The following supporting information can be downloaded at: https://www.mdpi.com/article/10.3390/polym14173503/s1, Figure S1: TGA curves of the P-0 film, GDAL plasticized films, and the GDAL plasticizer; Figure S2: DTG curves of the P-0 film, GDAL plasticized films, and the GDAL plasticizer; Figure S3: TGA curves of the P-0 film, isosorbide plasticized films, and the isosorbide plasticizer; Figure S4: DTG curves of the P-0 film, isosorbide plasticized films, and the isosorbide plasticizer; Figure S5: TGA curves of the P-0 film, films plasticized with PEG 200 and 400, and the pure PEG plasticizers; Figure S6: DTG curves of the P-0 film, films plasticized with PEG 200 and 400, and the pure PEG plasticizers; Figure S7: TGA curves of the P-0 film, films plasticized with PEG 600 and 100k, and the pure PEG plasticizers; Figure S8: DTG curves of the P-0 film, films plasticized with PEG 600 and 100k, and the pure PEG plasticizers; Figure S9: DSC first heating thermograms of the P-0 film and the GDAL plasticized films; Figure S10: DSC second heating thermograms of the P-0 film and the GDAL plasticized films; Figure S11: DSC first heating thermograms of the P-0 film and the isosorbide plasticized films; Figure S12: DSC second heating thermograms of the P-0 film and the isosorbide plasticized films; Figure S13: DSC first heating thermograms of the P-0 film and the films plasticized with PEG 200 and 400; Figure S14: DSC second heating thermograms of the P-0 film and the films plasticized with PEG 200 and 400; Figure S15: DSC first heating thermograms of the P-0 film and the films plasticized with PEG 600 and 100k; Figure S16: DSC second heating thermograms of the P-0 film and the films plasticized with PEG 600 and 100k; Figure S17: XRD diffractograms of the P-0 film and the GDAL plasticized films; Figure S18: XRD diffractograms of the P-0 film and the isosorbide plasticized films; Figure S19: XRD diffractograms of the P-0 film and the films plasticized with PEG 200 and 400; Figure S20: XRD diffractograms of the P-0 film and the films plasticized with PEG 600 and 100k.
