# Supplementary material for: Effect of Different Plasticizers on Thermal, Crystalline, and Permeability Properties of Poly(3–hydroxybutyrate–co−3–hydroxyhexanoate) Films
Source: Polymers (Basel). 2022 Aug 26;14(17):3503. doi: 10.3390/polym14173503 (PMC9460745; doi:10.3390/polym14173503)
Supplement: Supplementary file 1 [file polymers-14-03503-s001.zip › polymers-1851588-supplementary.pdf]

polymers

Supplementary material

Effect of different plasticizers on thermal, crystalline and permeability properties of Poly(3-hydroxybutyrate-co-3-hydroxyhexanoate) films

Yousof Farrag [1], Luis Barral [2], Oreste Gualillo [1], Danny Moncada [2], Belén Montero [2], Maite Rico [2] and Rebeca Bouza[2*]

[1]IDIS (Instituto de Investigación Sanitaria de Santiago), NEIRID Group (Neuroendocrine Interactions in Rheumatology and Inflammatory Diseases), Santiago University Clinical Hospital, Building C, Travesía da Choupana S/N, 15706 Santiago de Compostela, Spain.
[2] Universidade da Coruña, Grupo de Polímeros, Departamento de Física y Ciencias de la Tierra, Escuela Universitaria Politécnica, Serantes, Avda. 19 de Febrero s/n, 15471 Ferrol, Spain.
* Correspondence: rebeca.bouza@udc.es

Citation: Farrag, Y.; Barral, L.; Gualillo, O.; Moncada, D.; Montero, B.; Rico, M.; Bouza, R. Effect of Different Plasticizers on Thermal, Crystalline, and Permeability Properties of Poly (3-hydroxybutyrate-co−3-hydroxyhexanoate) Films. *Polymers* **2022**, *14*, 3503. https://doi.org/10.3390/polym14173503

Academic Editor: Beom Soo Kim

Received: 21 July 2022
Accepted: 23 August 2022
Published: 26 August 2022

Publisher's Note: MDPI stays neutral with regard to jurisdictional claims in published maps and institutional affiliations.

Copyright: © 2022 by the authors. Licensee MDPI, Basel, Switzerland. This article is an open access article distributed under the terms and conditions of the Creative Commons Attribution (CC BY) license (https://creativecommons.org/licenses/by/4.0/).

TGA and DTG curves of the PHBH films

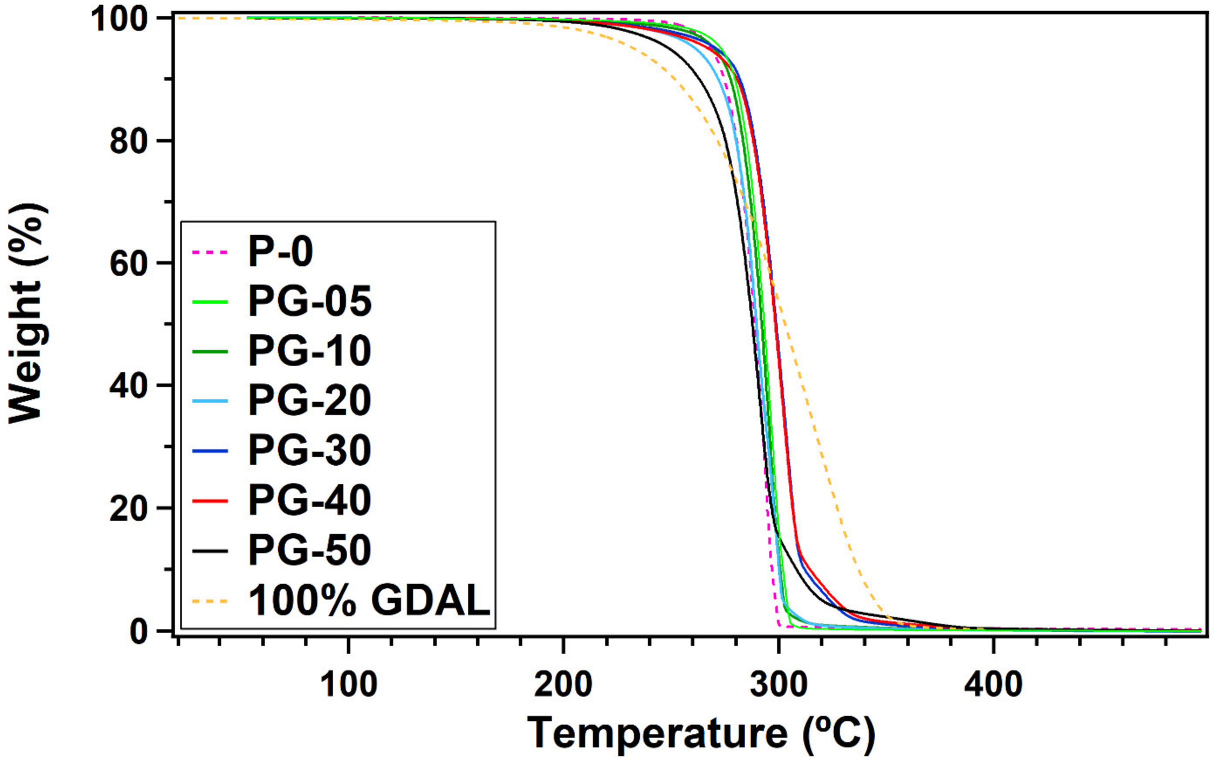

Figure S1. TGA curves of the P-0 film, GDAL plasticized films, and the GDAL plasticizer.

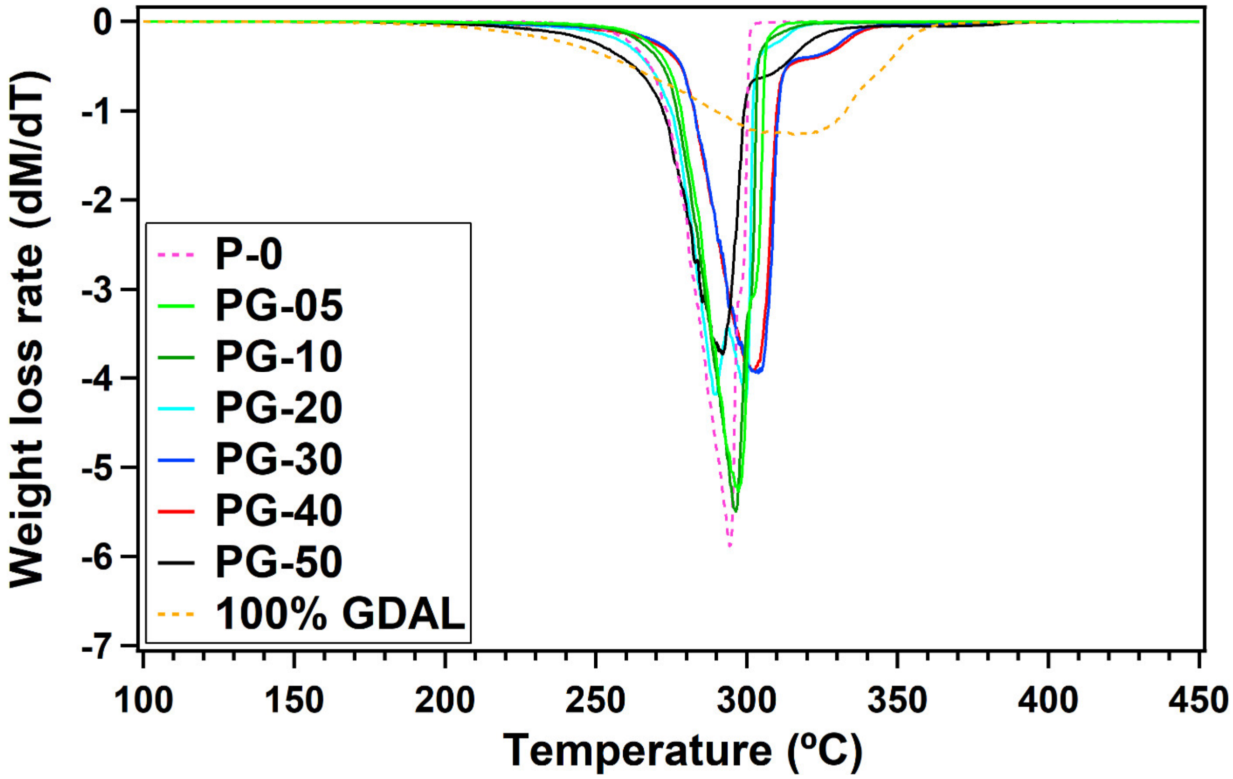

Figure S2. DTG curves of the P-0 film, GDAL plasticized films, and the GDAL plasticizer.

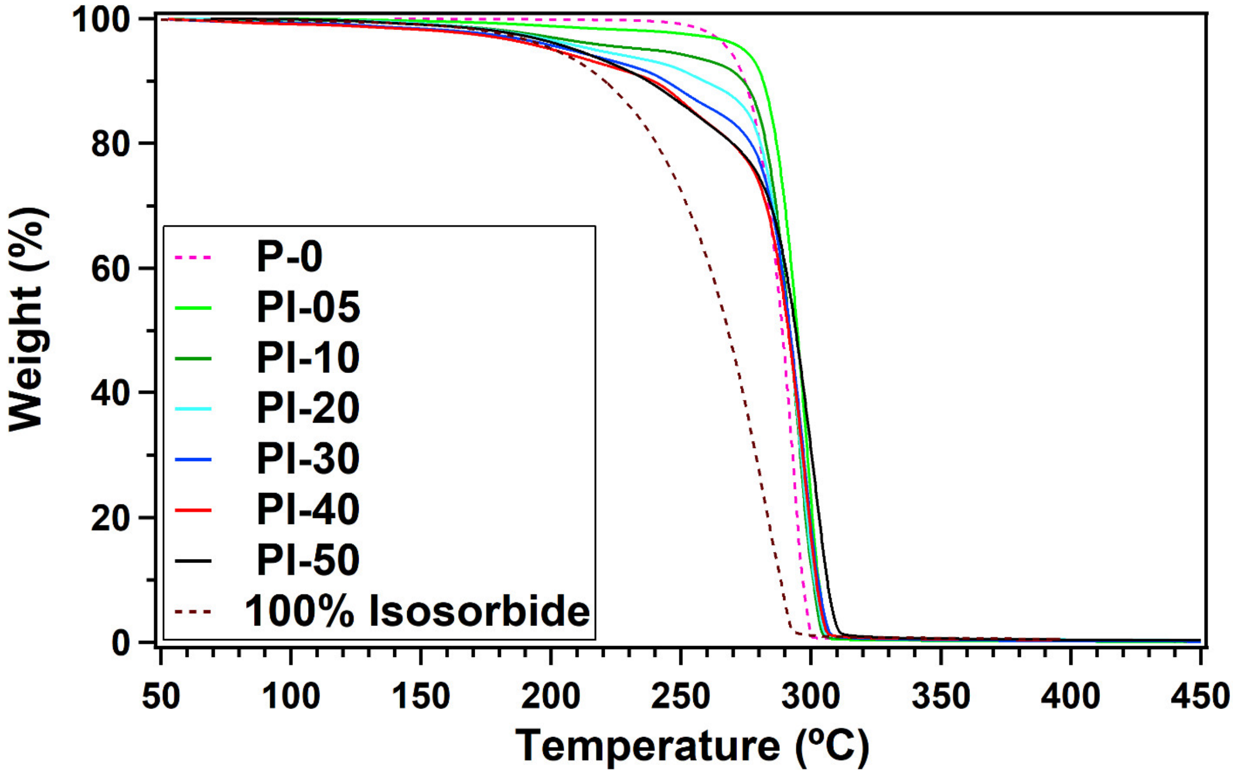

Figure S3. TGA curves of the P-0 film, isosorbide plasticized films, and the isosorbide plasticizer.

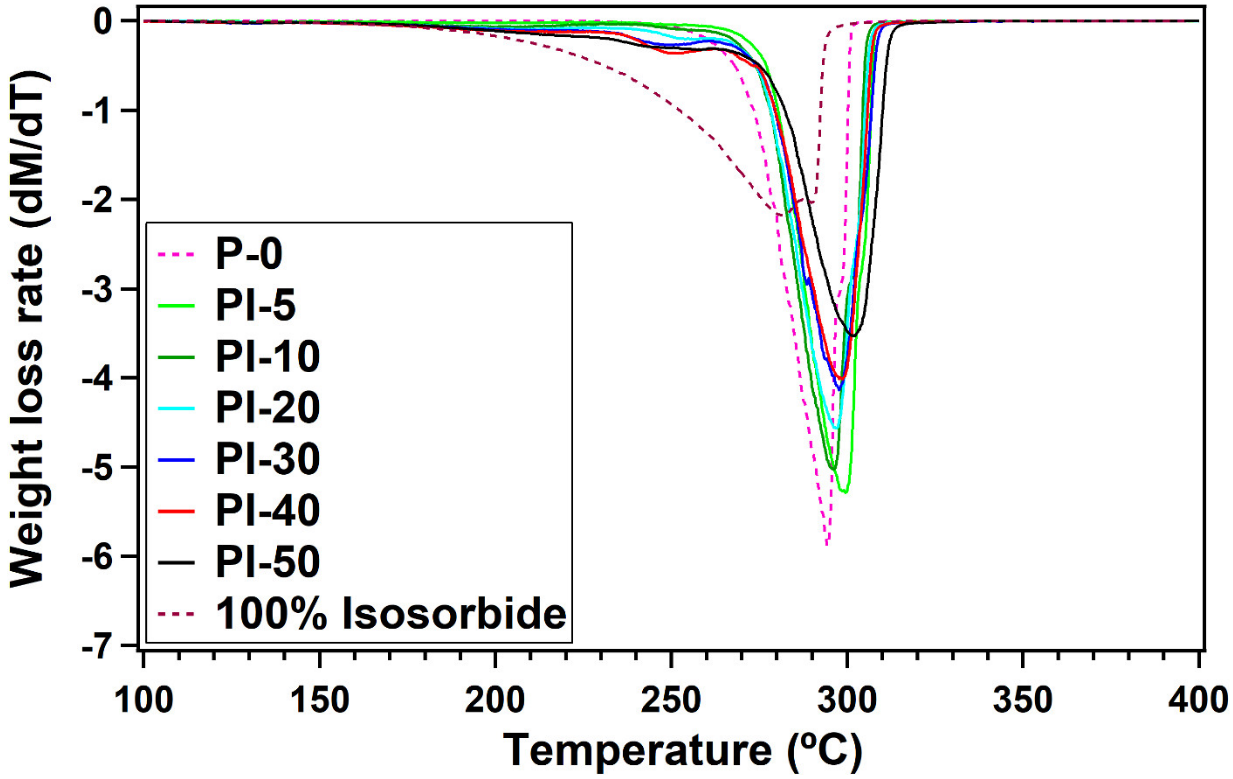

Figure S4. DTG curves of the P-0 film, isosorbide plasticized films, and the isosorbide plasticizer.

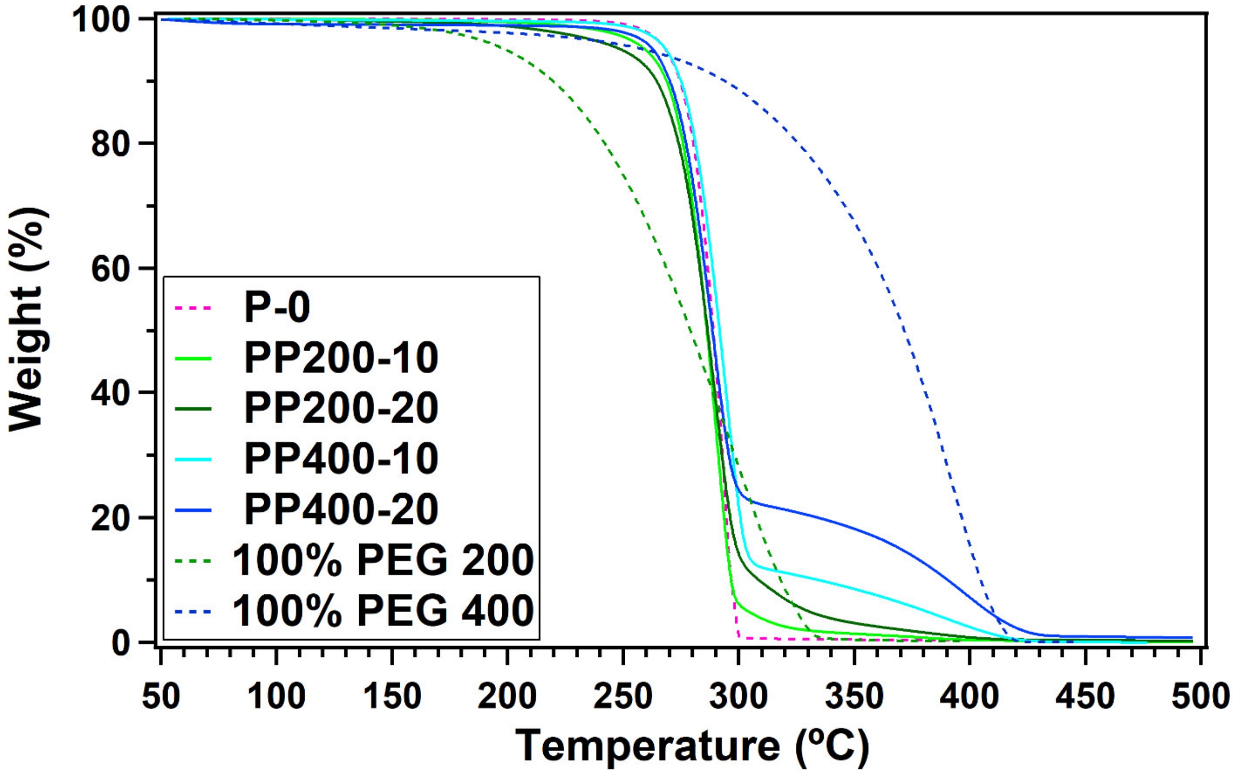

Figure S5. TGA curves of the P-0 film, films plasticized with PEG 200 and 400, and the pure PEG plasticizers.

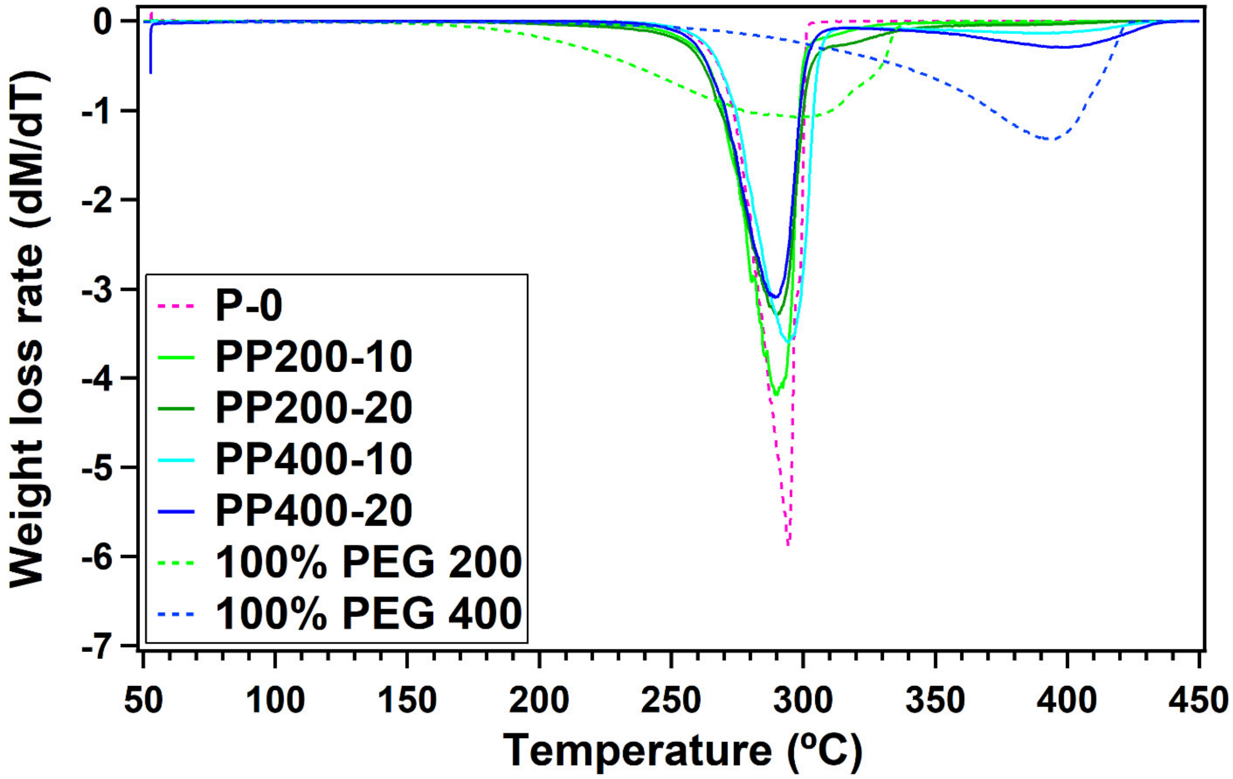

Figure S6. DTG curves of the P-0 film, films plasticized with PEG 200 and 400, and the pure PEG plasticizers.

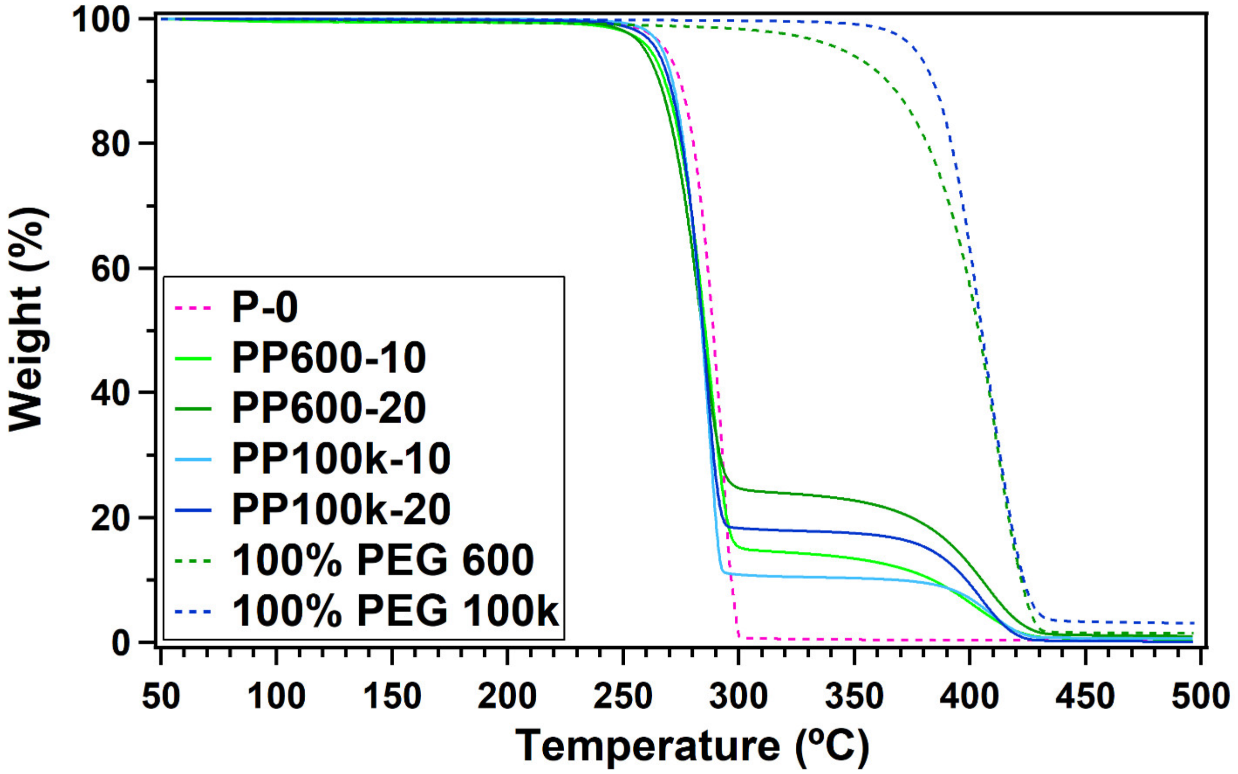

Figure S7. TGA curves of the P-0 film, films plasticized with PEG 600 and 100k, and the pure PEG plasticizers.

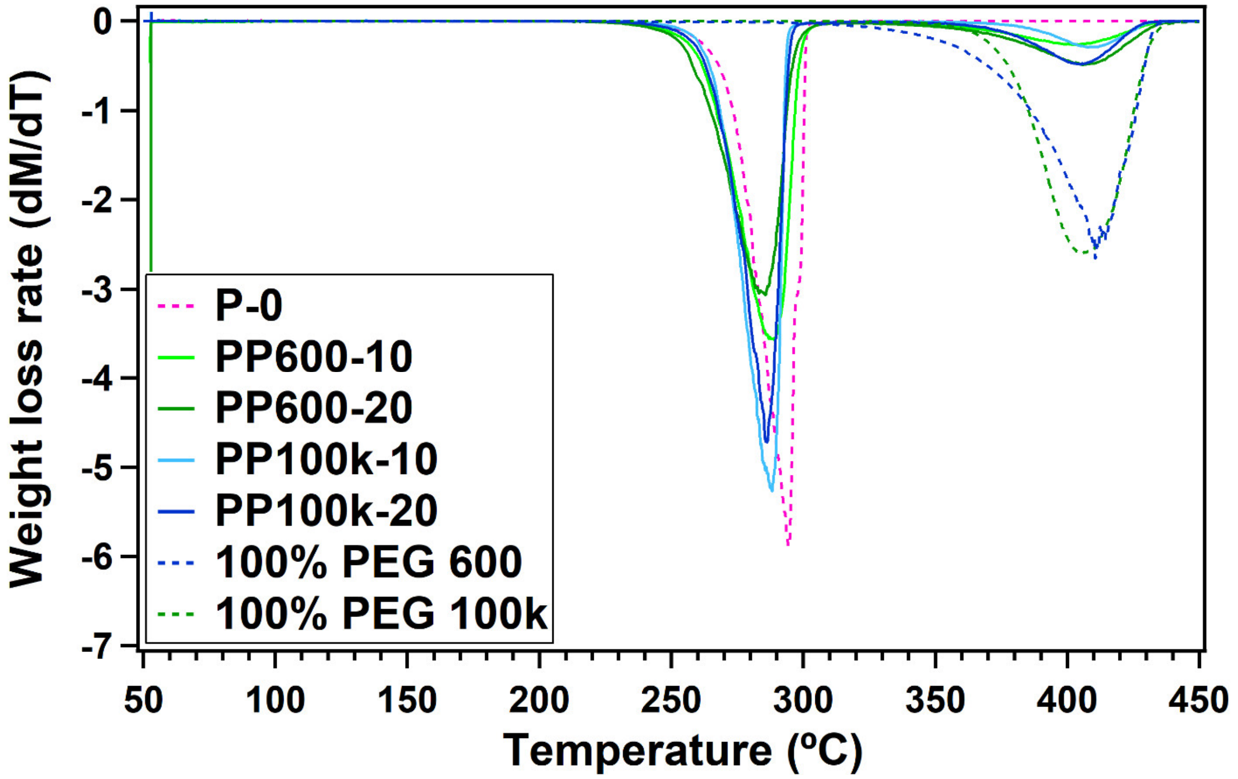

Figure S8. DTG curves of the P-0 film, films plasticized with PEG 600 and 100k, and the pure PEG plasticizers.

DSC thermograms of the PHBH films

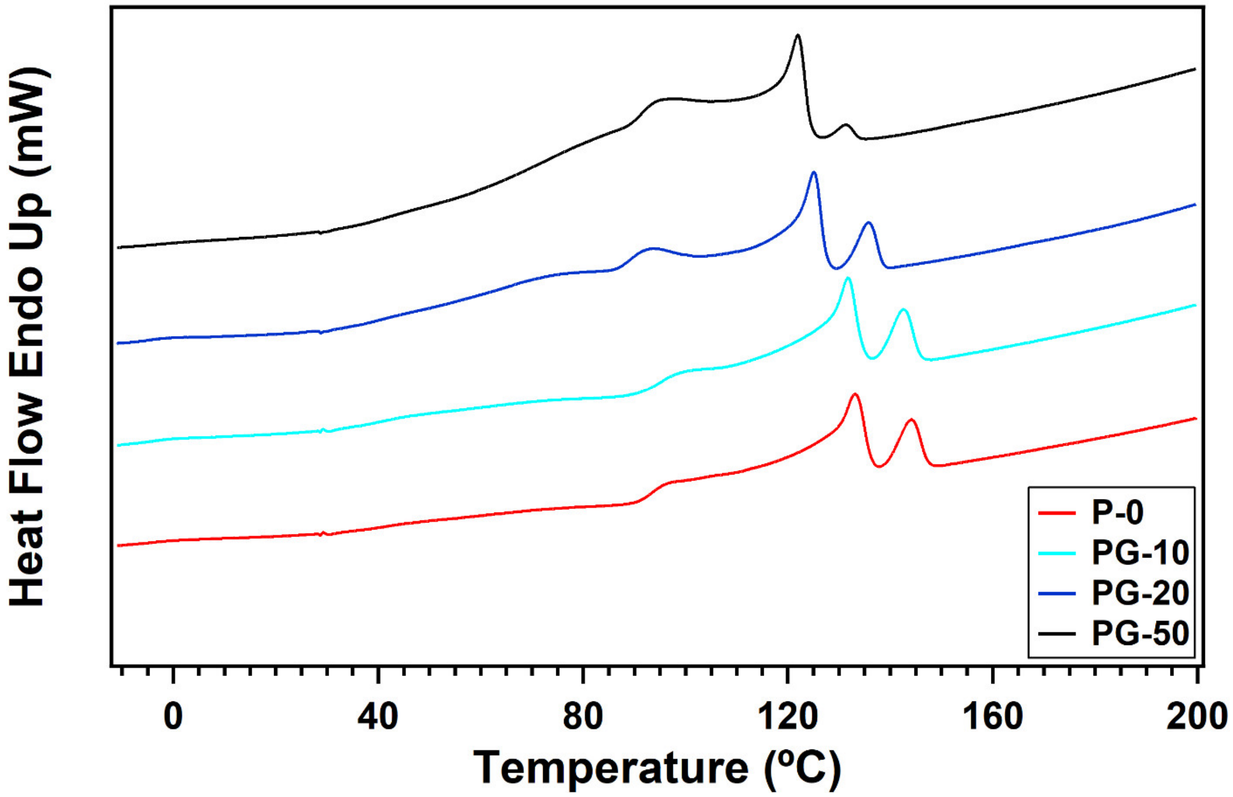

Figure S9. DSC first heating thermograms of the P-0 film and the GDAL plasticized films.

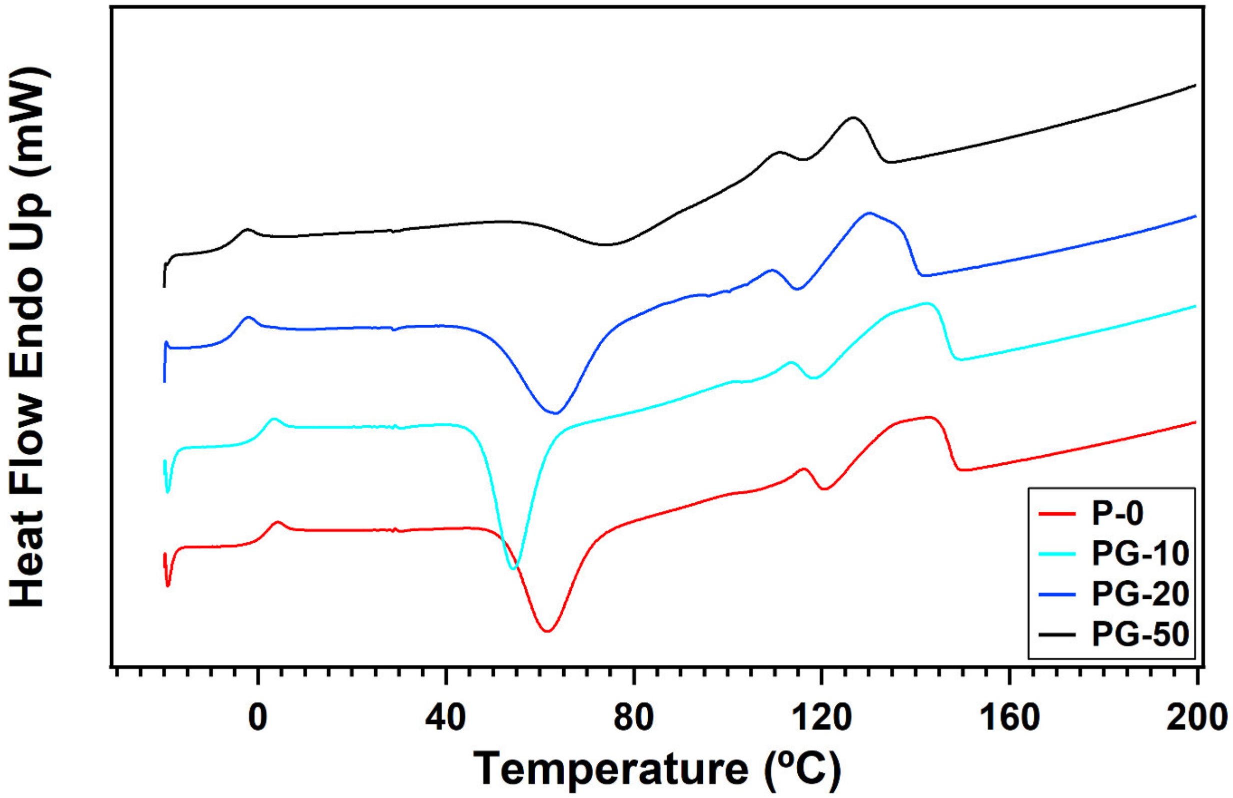

Figure S10. DSC second heating thermograms of the P-0 film and the GDAL plasticized films.

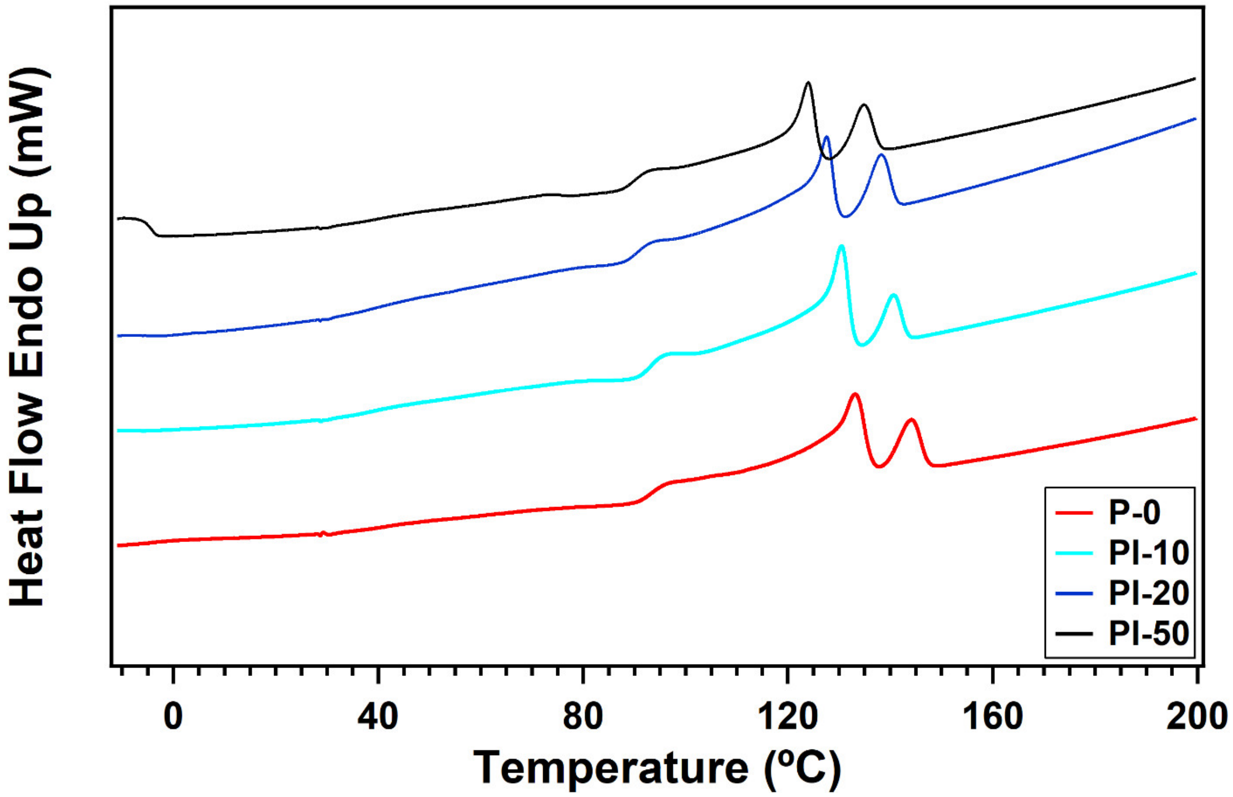

Figure S11. DSC first heating thermograms of the P-0 film and the isosorbide plasticized films.

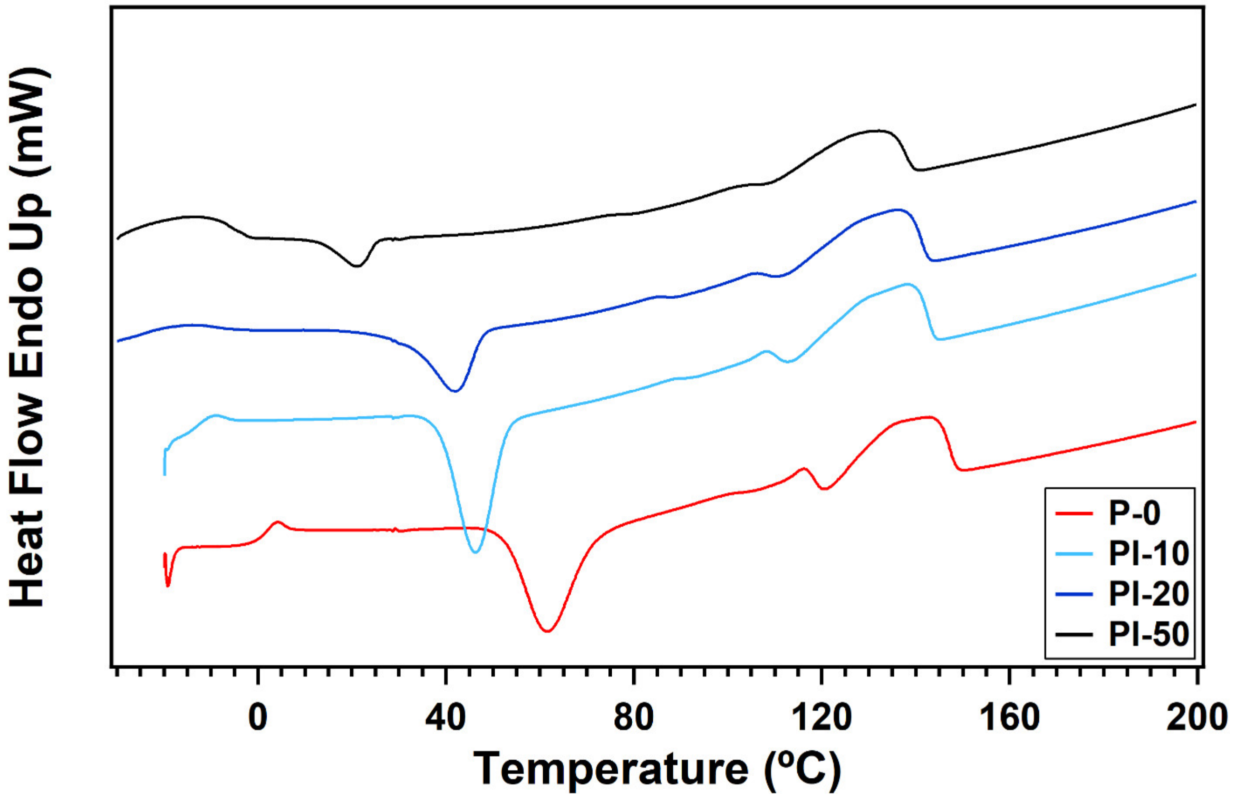

Figure S12. DSC second heating thermograms of the P-0 film and the isosorbide plasticized films.

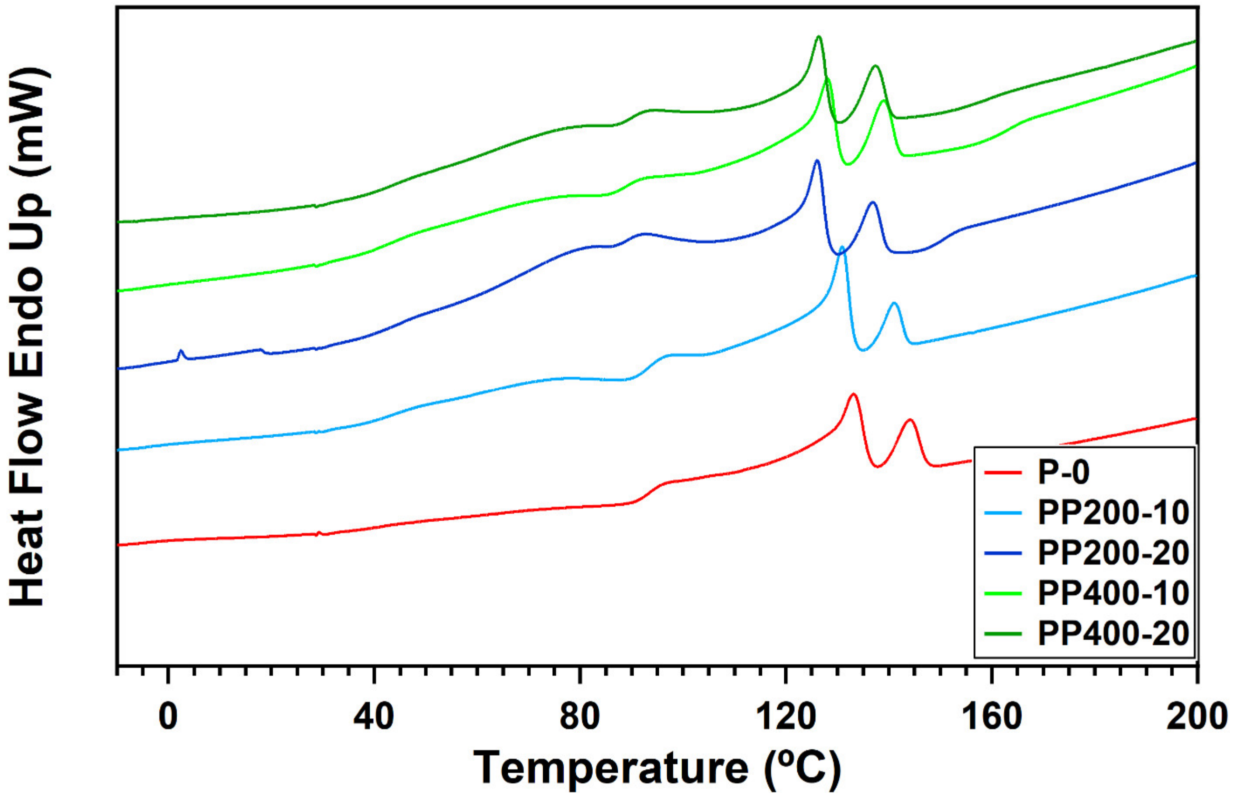

Figure S13. DSC first heating thermograms of the P-0 film and the films plasticized with PEG 200 and 400.

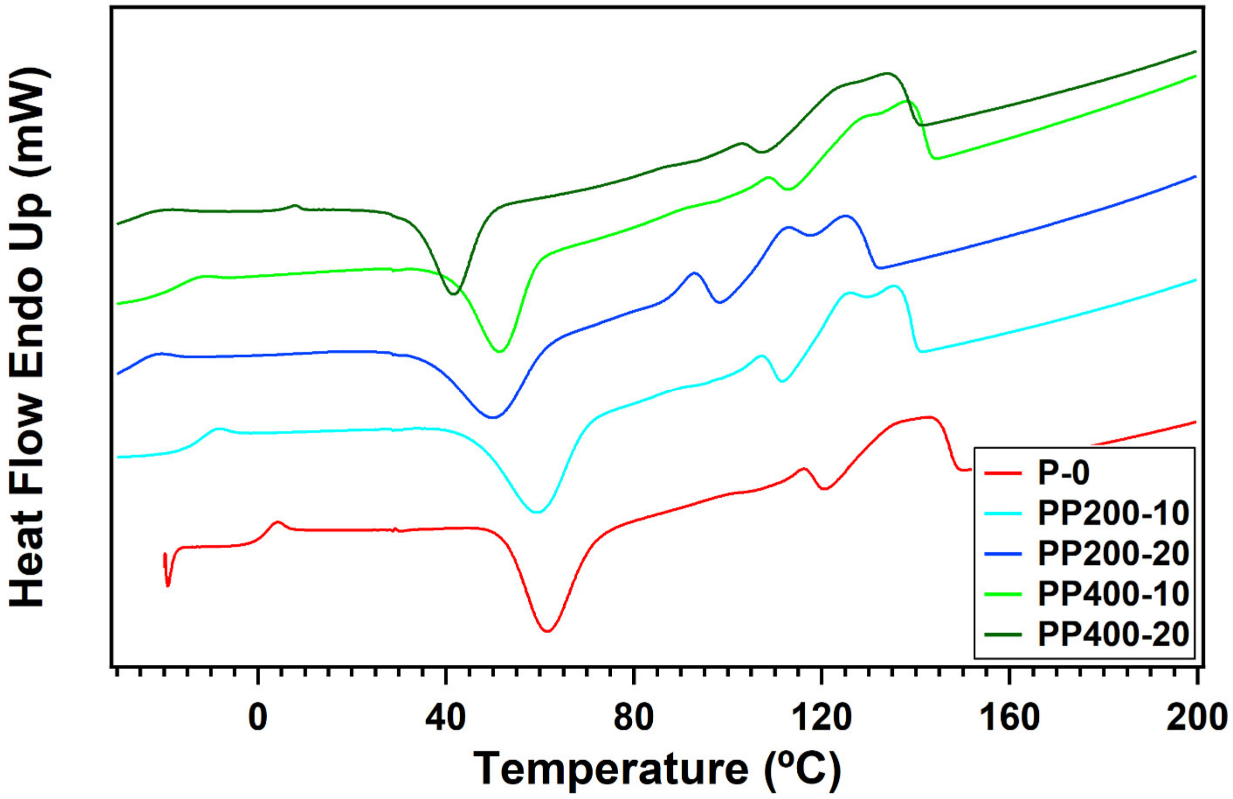

Figure S14. DSC second heating thermograms of the P-0 film and the films plasticized with PEG 200 and 400.

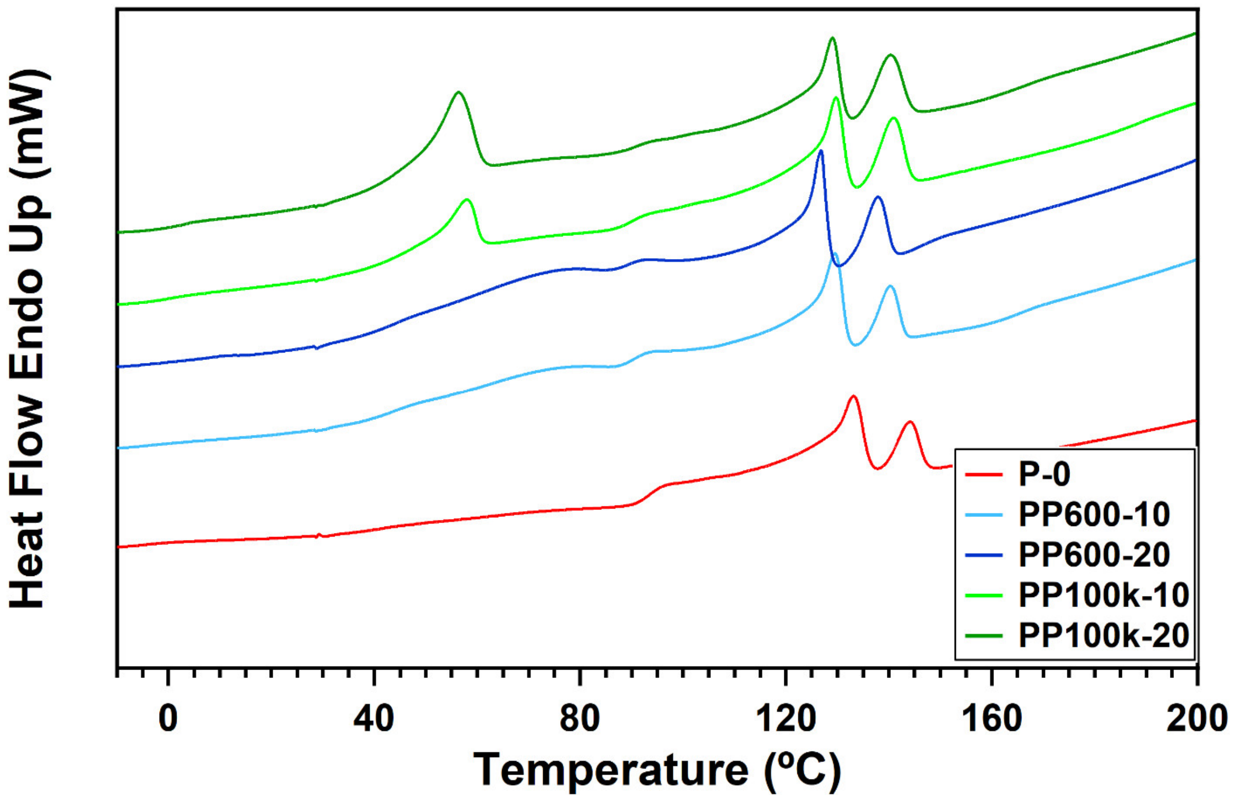

Figure S15. DSC first heating thermograms of the P-0 film and the films plasticized with PEG 600 and 100k.

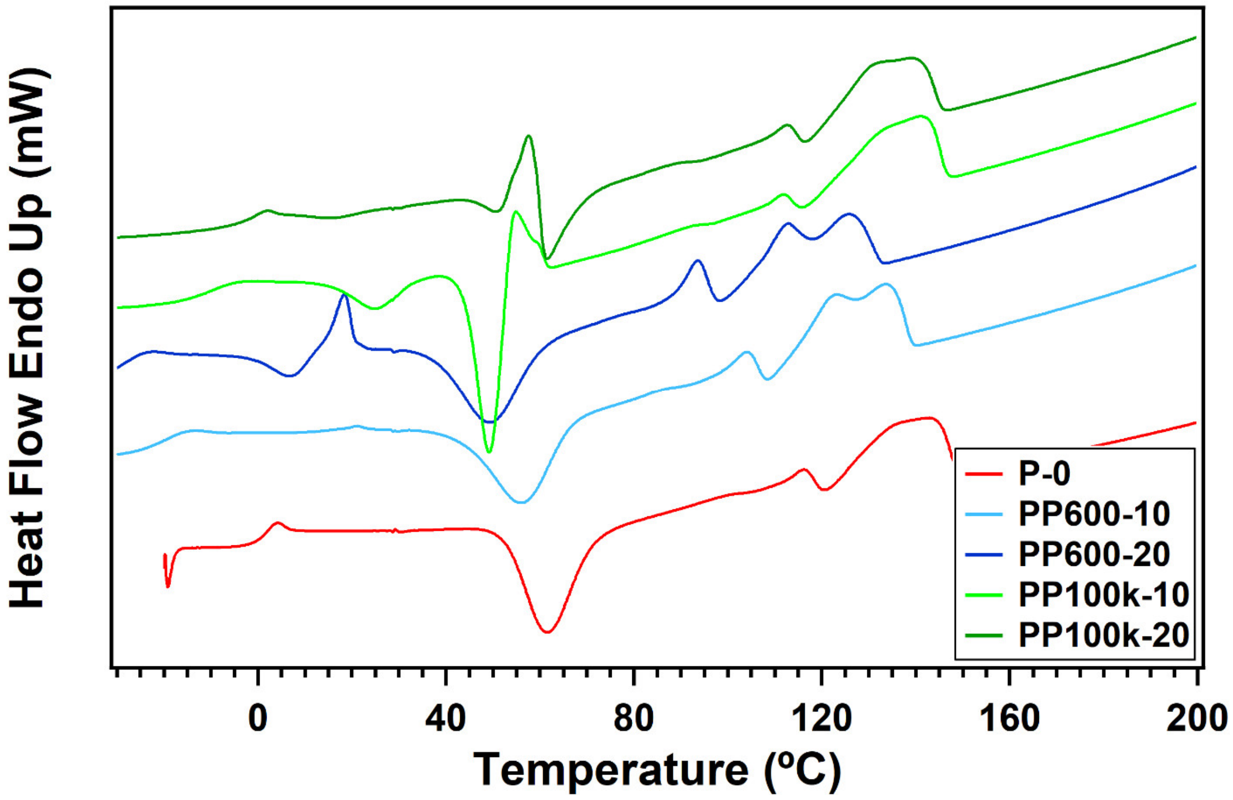

Figure S16. DSC second heating thermograms of the P-0 film and the films plasticized with PEG 600 and 100k.

XRD diffractograms of the PHBH films

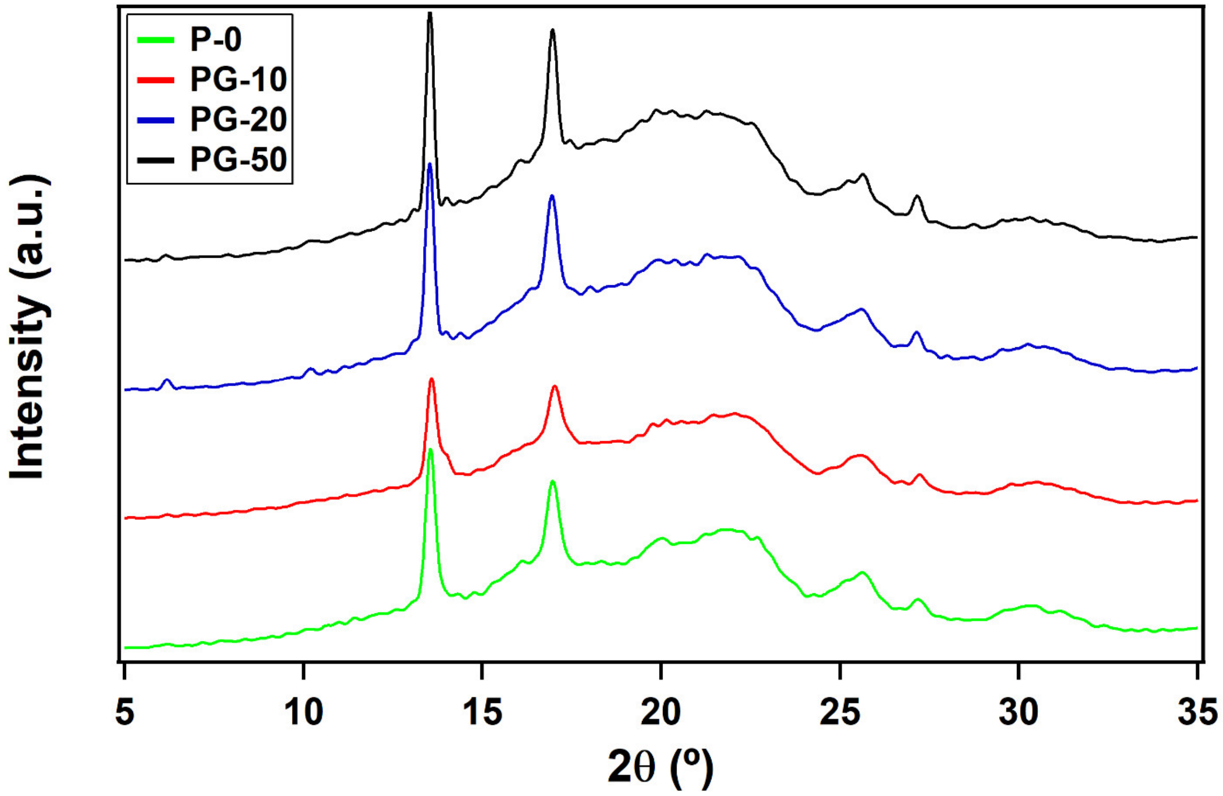

Figure S17. XRD diffractograms of the P-0 film and the GDAL plasticized films.

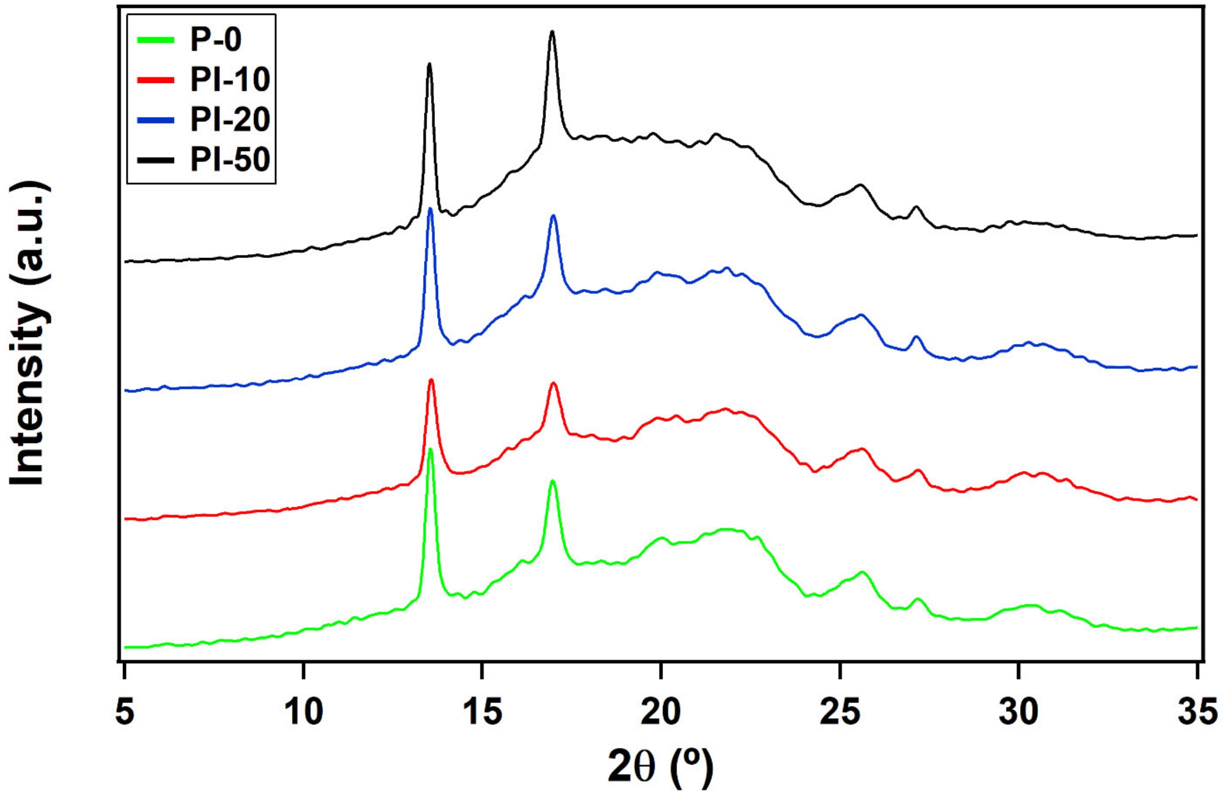

Figure S18. XRD diffractograms of the P-0 film and the isosorbide plasticized films.

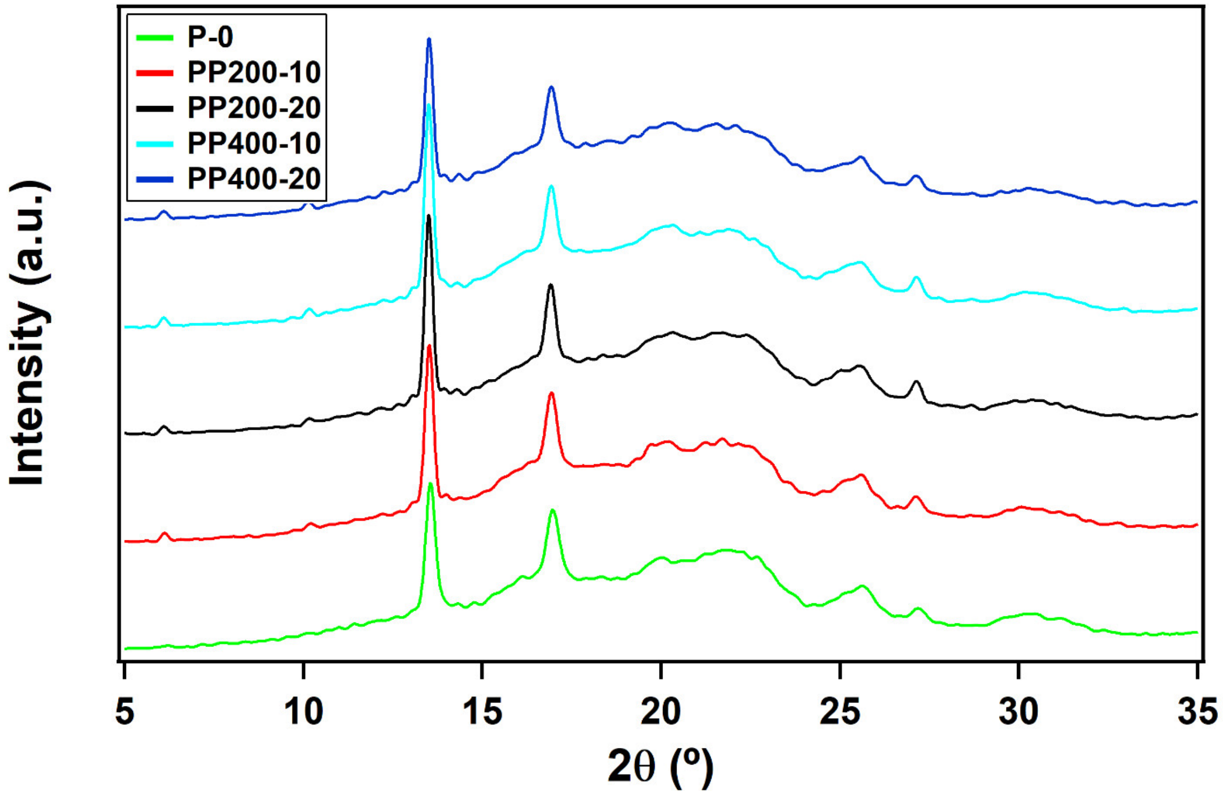

Figure S19. XRD diffractograms of the P-0 film and the films plasticized with PEG 200 and 400.

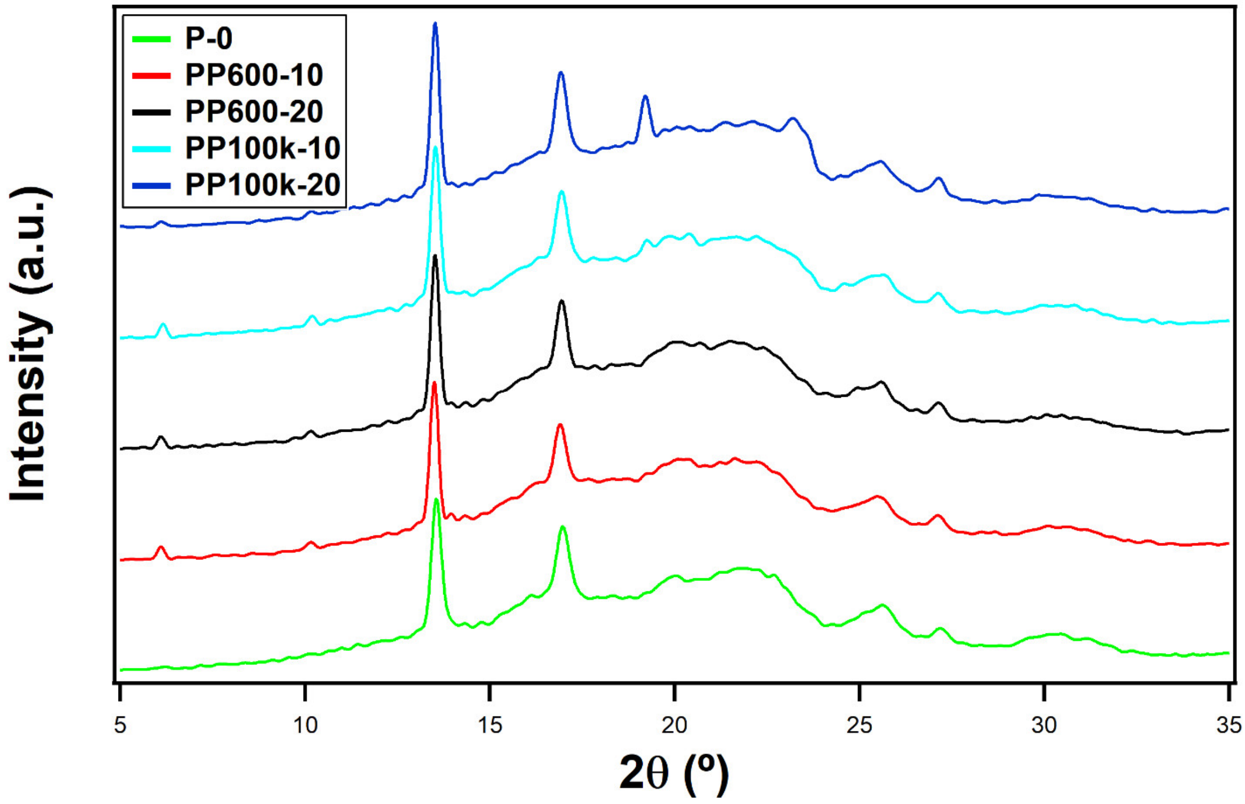

Figure S20. XRD diffractograms of the P-0 film and the films plasticized with PEG 600 and 100k.
